# Facilitating *Wolbachia* introductions into mosquito populations through insecticide-resistance selection

**DOI:** 10.1098/rspb.2013.0371

**Published:** 2013-06-07

**Authors:** Ary A. Hoffmann, Michael Turelli

**Affiliations:** 1 Departments of Genetics and Zoology, Bio21 Institute, The University of Melbourne, Parkville, Victoria 3010, Australia; 2 Department of Evolution and Ecology, University of California, Davis, CA 95616, USA

**Keywords:** symbiont, pesticide, disease vector, invasion, resistance

## Abstract

*Wolbachia* infections are being introduced into mosquito vectors of human diseases following the discovery that they can block transmission of disease agents. This requires mosquitoes infected with the disease-blocking *Wolbachia* to successfully invade populations lacking the infection. While this process is facilitated by features of *Wolbachia*, particularly their ability to cause cytoplasmic incompatibility, blocking *Wolbachia* may produce deleterious effects, such as reduced host viability or fecundity, that inhibit successful local introductions and subsequent spatial spread. Here, we outline an approach to facilitate the introduction and spread of *Wolbachia* infections by coupling *Wolbachia* introduction to resistance to specific classes of insecticides. The approach takes advantage of very high maternal transmission fidelity of *Wolbachia* infections in mosquitoes, complete incompatibility between infected males and uninfected females, the widespread occurrence of insecticide resistance, and the widespread use of chemical control in disease-endemic countries. This approach is easily integrated into many existing control strategies, provides population suppression during release and might be used to introduce *Wolbachia* infections even with high and seasonally dependent deleterious effects, such as the *w*MelPop infection introduced into *Aedes aegypti* for dengue control. However, possible benefits will need to be weighed against concerns associated with the introduction of resistance alleles.

## 1. Introduction

There is increasing interest in using *Wolbachia* bacterial infections to suppress mosquito-transmitted diseases. This follows the successful introduction of *Wolbachia* into disease vectors, particularly *Aedes aegypti* Linnaeus, 1762 [1] and *Aedes albopictus* Skuse, 1894 [2], and the realization that *Wolbachia* act as natural agents to suppress disease [3,4]. Several experiments have shown that *Wolbachia* can suppress dengue, chikungunya, yellow fever and other diseases [5], and that they might even be effective against other diseases including malaria [6]. Maternally inherited *Wolbachia* possess several characteristics that facilitate their invasion and rapid spread into natural populations [7], particularly their ability to cause cytoplasmic incompatibility that leads to embryo death when uninfected females mate with infected males.

Preliminary field trials on *A. aegypti* infected with the *w*Mel *Wolbachia* have demonstrated successful invasion of two sites in northern Australia [8]. This infection appears stable and is present at a frequency approaching 100 per cent at these sites more than two years after the invasion was initiated (I. Iturbe-Ormaetxe 2013, unpublished data). However, this infection has relatively minor deleterious effects [1], facilitating its establishment because there is a relatively low unstable equilibrium point that has to be exceeded for invasion. Other *Wolbachia* strains with the potential to provide stronger blockage of disease transmission may have much larger deleterious effects, making initial invasion and particularly subsequent spatial spread difficult or impossible [9]. In particular, the *w*MelPop infection provides complete blockage of dengue [5], but *A. aegypti* mosquitoes with this infection suffer significantly reduced fecundity and egg hatch, particularly when eggs are in a dry quiescent state [10,11]. This feature makes it more difficult to introduce *w*MelPop into populations as reflected by its rate of increase in semi-natural population cages when compared with *w*Mel [1]. Moreover, successful invasion of *w*MelPop into natural populations is likely to require releasing many infected mosquitoes to overcome the higher unstable equilibrium point [10]. Whereas *w*Mel successfully invaded populations over three months when releases increased natural adult populations by 1.5–2 times [8], local invasions by infections such as *w*MelPop over a similar time scale are likely to require introduction rates leading to a transient increase in adult population size of greater than twofold. The resultant increase in mosquito biting rate may become unacceptable to local community members particularly in disease-endemic areas. Once introduced into a local area, the infection may be lost following mosquito migration from surrounding uninfected populations [9] or during the dry season when *w*MelPop imposes a substantial fitness cost [10].

To counter these issues, a method is needed to facilitate the spread of *Wolbachia* that is consistent with current control methods and acceptable in countries where diseases are endemic. One possibility is to integrate *Wolbachia* releases with pesticide applications by introducing insecticide resistance into the *Wolbachia*-infected line. Resistance is widespread in mosquitoes including disease vectors such as *A. aegypti*, and resistance evolution has led to chemical methods of control becoming ineffective and being abandoned in some instances [12,13]. At first sight, this strategy might seem doomed to failure because any association between the nuclear-based resistance alleles and the maternally inherited *Wolbachia* in the released strain is expected to break down rapidly after *Wolbachia* are introduced [14]. However, as we argue below, there are unique features of mosquito–*Wolbachia* infections that make this strategy attractive, providing a potential path for introducing *Wolbachia* into local populations more easily and without increasing mosquito populations. The strategy could also help secure the persistence of infections such as *w*MelPop across a dry season and assist their spatial spread, as long as the unstable equilibrium (the frequency of *Wolbachia* in a population that needs to be exceeded for the *Wolbachia* to spread to fixation) is not prohibitively high [9].

## 2. Approach

We assume that resistance can be selected in a mosquito population infected by *Wolbachia*. In *A. aegypti*, there is no evidence that *Wolbachia* infections directly influence resistance to commonly used chemicals [15]. By contrast, selection on the nuclear genome of mosquitoes can readily increase resistance to a range of pesticides including organophosphates, pyrethroids and organochlorines [12,16]. Resistant *Wolbachia*-infected populations might be established through a variety of means. Resistance could be identified in field populations, and then introduced into infected strains by backcrossing to the field populations, particularly when combined with ongoing screening for resistance, in the same way as *Wolbachia* strains have been backcrossed to introduce the background of a target natural population prior to release [10]. Selection for resistance could also take place within the background of an infected population by applying a laboratory-based method for increasing resistance [16]. Finally a resistant population could be developed independently in the laboratory through selection or mutagenesis, and *Wolbachia* could be subsequently introduced into this population through backcrossing. With these approaches, it should be possible to establish infected populations resistant to different chemicals on various genetic backgrounds, with the resistant phenotype maintained through regular exposure to discriminatory doses.

Potential targets for resistance screening are the chemicals widely applied for mosquito control, such as organophosphates applied to larval breeding sites, or pyrethroid and organophosphate adulticides used in fogging buildings and surrounding areas [17]. Resistance to these chemicals has been detected in some *Aedes* mosquito populations [12,13], making it feasible to select for resistance or identify natural populations with resistance. It should also be possible to develop resistance to chemicals or formulations that are not used for routine mosquito control, a strategy that might be acceptable to the community and to regulatory authorities.

Once infected populations resistant to chemicals have been developed, they can be introduced into natural populations through releases. Any disequilibrium between *Wolbachia* and its nuclear background is expected to be roughly halved each generation when the infection is being introduced [18]. Under imperfect maternal transmission of the *Wolbachia* infection and incomplete cytoplasmic incompatibility (CI)—the situation for the *w*Ri infection of *Drosophila simulans* (Sturtevant), which has been extensively characterized [19]—uninfected individuals will arise with the nuclear background of the infected population and vice versa. However, in naturally infected populations of *Culex* and *Aedes* mosquitoes, the failure rate of maternal transmission, **μ**, is 0 or very near 0, and cytoplasmic incompatibility is complete or very nearly complete [20,21]. This means that whereas matings between infected males and uninfected females do not produce any offspring, the progeny of infected females are always viable and infected. Moreover, in lines of artificially infected *Aedes* mosquitoes, it also appears that **μ** = 0 and there is complete or near-complete incompatibility [1,10]. Thus, in these infections the association between *Wolbachia* infection and nuclear-resistance alleles is expected to break down only in one direction, with infected individuals acquiring susceptibility alleles through mating with uninfected males, but no transfer of resistance alleles from the release stock to the *Wolbachia*-uninfected component of the mosquito population.

The potential impact of resistance on *Wolbachia* invasions can be illustrated by considering two aspects of *Wolbachia* releases, the position of the unstable point for invasion and the speed at which invasion can take place.

To explore potential effects of coupling resistance alleles to *Wolbachia* introductions on unstable points, we present idealized analyses aimed at approximating the quantitative effects rather than capturing specific biological details. To simplify the algebra, we assume discrete generations and that *Wolbachia* affects female fecundity, with infected females having relative fecundity *F* = 1 − *s*_f_, with *s*_f_ > 0. As noted below, the parameter *F* can also approximate viability effects attributable to *Wolbachia*. We assume perfect maternal *Wolbachia* transmission and complete CI (as seen for the *w*MelPop and *w*Mel infections—[1,10,11]) and for simplicity we also assume that insecticide resistance is governed by a single diallelic locus, with alleles denoted R and S (see [12] for examples of single loci contributing significantly to insecticide resistance, although we acknowledge that resistance based on detoxification in particular is likely to have a more complex genetic basis in *Aedes* [13]). When insecticide is applied, the three genotypes (RR, RS, SS) have relative viabilities (1, 1 − *hs*, 1 − *s*), where *s* > 0 and *h* describes the dominance of insecticide susceptibility, with 0 ≤ *h* ≤ 1. We assume no interaction between the effects of *Wolbachia* and insecticide resistance. The resistance allele, R, is assumed to be fixed in the infected release population, but initially rare or absent in the uninfected target populations. This reflects the ability to artificially select and/or backcross to produce a resistant population [16], and the range of resistance levels typically seen in uninfected populations [22–24]. If R is not entirely fixed within the released populations, the quantitative results are largely unaffected, because as soon as *Wolbachia*-infected females mate with local males (who are predominantly SS), there will be an appreciable frequency of S within the resulting *Wolbachia*-infected offspring. We assume random mating, irrespective of infection status or resistance genotype.

Under these assumptions, the frequency dynamics of the *Wolbachia* infection and the resistance allele are described by three variables: *p_t_*, the infection frequency among adults in generation *t*, *r*_U,*t*_, the frequency of R among uninfected adults, and *r*_I,*t*_, the frequency of R among infected adults. To obtain the frequencies in the next generation, we describe in turn the effects of random mating, CI (with a fecundity deficit for infected females), and viability selection. Let *f*(RR,U)*_t_*_+1_ (*f*(RR,I)*_t_*_+1_) denote the frequency of uninfected (infected) RR individuals among the viable zygotes in generation *t* + 1. Note that viable uninfected zygotes are produced only by mating between uninfected adults, whereas infected zygotes are produced by infected mothers mating with either infected or uninfected fathers. Because of differences in the allele frequencies between infected and uninfected individuals, the genotype frequencies among the infected zygotes will not be in Hardy–Weinberg proportions. The frequencies for viable zygotes are2.1a
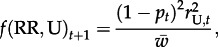
2.1b
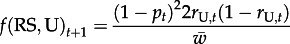
2.1c
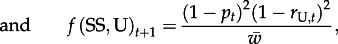
whereas2.2a
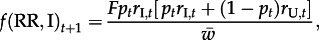
2.2b
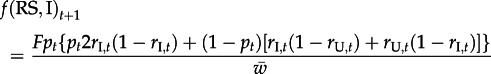
and2.2c
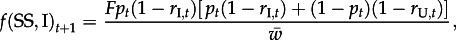
with2.3
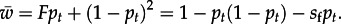
Note that the right-hand side of each equation in (2.2) has two terms, corresponding to infected females mating with either infected or uninfected males. The leading term *F* on the right sides of (2.2) reflects the reduced fecundity of infected females. This multiplicative term can also incorporate viability differences produced by the *Wolbachia* infection. Expression (2.3) for 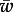 shows that the expected number of viable zygotes is reduced by both CI and the fecundity deficit of infected females.

To obtain the genotype frequencies among adults, denoted *F*(RR,I), etc., we apply viability selection to these six classes of zygotes to obtain2.4a
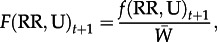
2.4b
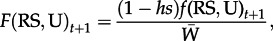
2.4c
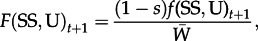
2.4d
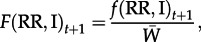
2.4e
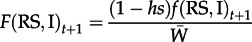
and2.4f
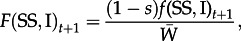
with2.5
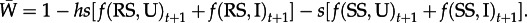
To complete the recursions, we calculate the allele and infection frequencies among the adults:2.6a
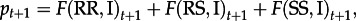
2.6b
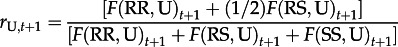
and2.6c
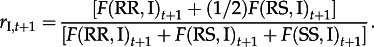
Because we have complete CI and we are interested in strong selection in favour of resistance, there are no simple analytical approximations for the dynamics. However, the qualitative behaviour of the system can be easily understood. In particular, if the resistance allele is initially absent from the uninfected population (*r*_U,0_ = 0), it will remain absent because of complete CI and faithful maternal transmission of *Wolbachia*. By contrast, mating of infected females with uninfected males effectively produces unidirectional migration of susceptibility alleles from the uninfected population to the infected population. These alleles are steadily eliminated by natural selection on a time scale proportional to 1/*s*. When there is no genetic variation at the resistance locus, we have the simple CI dynamics described by the Caspari & Watson [25] model with *s*_h_ = 1. Hence, 0 and 1 are stable equilibrium frequencies separated by an unstable equilibrium at 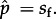 If the infected individuals all carry the resistance allele and insecticide is applied, the unstable point will be effectively reduced to 0 if *F*(1 + *s*) ≥ 1 and will be approximately2.7
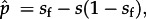
when *F*(1 + *s*) < 1. As susceptibility alleles are introduced into the infected population from matings with uninfected males, the unstable equilibrium rises. Indeed, if the initial infection frequency is 0.5 or less, with the infected population nearly fixed for the resistance allele but the resident population almost all susceptible, the resistance allele frequency among the infected F_1_ generally drops by at least 50 per cent. However, this can be countered by ongoing releases of resistant, infected individuals; ongoing releases are a feature of recent successful attempts to invade *Wolbachia* into uninfected mosquito populations [8]. We consider single and multiple releases in turn.

### (a) Single release analysis

For simplicity, we initially analyse a single release into an isolated population and assume that the fecundity deficit, *s*_f_, for *Wolbachia*-infected individuals is 0.3 (or 0.4) in line with empirical estimates for the *w*MelPop infection [10]. Hence, without introducing pesticide resistance, the initial infection frequency would have to exceed 0.3 (0.4) for the infection to spread. Because the goal is relatively rapid local population transformation, we ask what minimum initial frequency, denoted Min *p*_0_, is required for the infection frequency to exceed 0.95 within 20 generations. Without resistance selection, if *s*_f_ = 0.3, Min *p*_0_ = 0.303. This differs little from 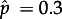 because complete CI rapidly drives the infection into the population. With *s*_f_ = 0.4, Min *p*_0_ = 0.401 (versus 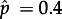). We suppose that all the released infected individuals are homozygous for the resistance allele (RR), and initially assume that all residents are SS but then allow for a low level of resistance in the pre-release target population.

**Figure 1.**
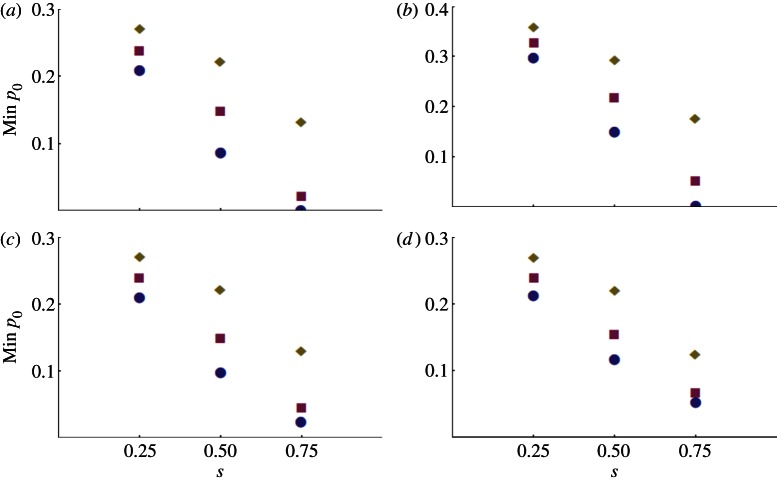
Minimum value of *p*_0_ that produces *Wolbachia* frequency *p_t_* = 0.95 within 20 generations, assuming *r*_I,0_ = 1. In each panel, selection against susceptibility (*s*) varies as does the dominance of susceptibility (*h*: 0.9, diamonds; 0.5, squares; 0.1, circles). (*a*,*b*) Assume no insecticide resistance in the target population (*r*_U,0_ = 0), and *s*_f_ = 0.3 (*a*) or *s*_f_ = 0.4 (*b*). (*c*,*d*) Investigate the effects of a low level of insecticide resistance in the target population: *r*_U,0_ = 0.001 (*c*) or *r*_U,0_ = 0.01 (*d*). (Online version in colour.)

Figure 1 illustrates how Min *p*_0_ varies as a function of *s*, the intensity of selection against susceptibility; *h*, the dominance of susceptibility (*h* = 0 corresponds to the susceptibility allele (S) being recessive, i.e. resistance is dominant); and *r*_U,0_, the frequency of insecticide resistance in the pre-release target population. The diamonds correspond to *h* = 0.9 (resistance nearly recessive), the squares correspond to *h* = 0.5 (additive effects) and the circles correspond to *h* = 0.1 (resistance nearly dominant). Panels (*a*,*b*) assume that the target population initially has no resistance alleles (*r*_U,0_ = 0), whereas panels (*c*,*d*) illustrate the consequence of low levels of resistance in the target population, as might be expected at mutation-selection equilibrium [26]. When *s* is less than *s*_f_, as illustrated by the leftmost points in each panel of figure 1, with *s* = 0.25, the co-introduction of resistance alleles with *Wolbachia* has relatively little effect on Min *p*_0_, irrespective of the level of dominance. As expected, introducing resistance alleles that are more dominant (*h* = 0.1, blue circles) has a larger effect. By contrast, when selection against susceptibility exceeds the cost of *Wolbachia* infection (*s* > *s*_f_), co-introduction of resistance and *Wolbachia* can have a large effect. With very strong insecticide-induced selection (*s* = 0.75), the role of dominance is critical. If resistance is nearly recessive (i.e. susceptibility nearly dominant, *h* = 0.9), Min *p*_0_ is roughly halved, regardless of whether resistance is already segregating in the target population. However, if heterozygotes suffer at least half the fitness loss of susceptible homozygotes, Min *p*_0_ is greatly reduced. Estimates of *s* in field populations of mosquitoes across a region are relatively low [27]. However, for the *Wolbachia* releases we are not concerned with selection on resistance alleles across a large area. Instead we envisage targeted pesticide applications with the potential to kill most individuals of a life cycle stage, the type of approach used to eradicate invasive incursions of populations of mosquitoes [28] and akin to repeated fogging treatments that can kill almost all of the mosquitoes at a particular life stage [e.g. 29].

Figure 1*c*,*d* shows that there can be a complex interaction between Min *p*_0_ and *r*_U,0_, the initial frequency of resistance in the target population. Intuitively, larger *r*_U,0_ might be expected to generally impede the efficacy of co-introducing resistance and *Wolbachia*. However, when resistance is initially introduced, its frequency among the infected individuals plummets as the infected females mate with resident males. Simultaneously, strong selection favouring resistance increases *r*_U,t_. Even when resistance is rare, its presence among uninfected males can slightly raise the resistance frequency among infecteds, *r*_I,*t*_, above what it would be if *r*_U,0_ = 0. As a result, the extremely small values of Min *p*_0_ predicted when *r*_U,0_ = 0 are not robust to even low levels of initial resistance in the target population (panel (*d*), *r*_U,0_ = 0.001). Figure 1 illustrates that Min *p*_0_ depends on several parameters that will be difficult to estimate in the field. Nevertheless, if very strong selection in favour of resistance can be imposed, the co-introduction of *Wolbachia* and semi-dominant resistance will generally reduce the threshold introduction frequency (Min *p*_0_) by at least a factor of five.

### (b) Multiple release analyses

The quantitative effects of dominance and resistance selection are more realistically captured by considering conditions for *Wolbachia* establishment with repeated releases of *Wolbachia*-infected, insecticide-resistant mosquitoes. Modelling repeated releases is complicated by the fact that *A. aegypti* females rarely remate. Thus, adult females released are likely to have already mated rather than mating with resident males, and the frequency of incompatible and compatible matings is not set by the frequency of *Wolbachia* in the population.

To approximate the consequences of repeated releases, we assume that the adults released in generation *t* contribute a fraction *m* of the viable zygotes in generation *t* + 1. As in figure 1, we assume that all released individuals are *Wolbachia*-infected and homozygous for the resistance allele (RR), whereas initially almost all residents are SS. To model this, we simply insert recursions to describe immigration between (2.2) and (2.4). To the zygotes produced by field-born *A. aegypti*, we add those produced by the already-mated females released. These zygotes are all *Wolbachia*-infected and homozygous for the resistance allele. Letting *f′*(RR,I)*_t_*_+1_ denote the frequency after immigration, we have the recursions2.8a
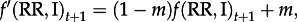
2.8b
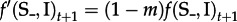
and2.8c
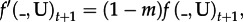
which reflect the fact that only (RR,I) individuals are released. In (2.8*b*), *f′*(S_,I) denotes *f′*(SS,I) or *f′*(SR,I); in (2.8*c*), *f′*(_,U) denotes *f′*(SS,U), *f′*(SR,U) or *f′*(RR,U).) We now use the *f′*(RR,I)*_t_*_+1_, etc., in place of *f*(RR,I)*_t_*_+1_, etc., in (2.4) and (2.5) to obtain the adult frequencies that enter (2.6).

We address two questions. First, what is the minimum per-generation introduction rate (measured as *m*, the fraction of the population of viable zygotes produced by the adult releases in the previous generation) that will produce a 95% infection rate within 20 generations? We denote this critical migration rate *m*_c_. Second, we recalculate the critical migration rate assuming that releases can be performed for only ten generations. Obviously, this second critical migration rate, denoted *m*_c(10)_, must be higher than *m*_c_.

Similar questions were addressed numerically by Hancock *et al.* [30], who used a density-dependent population model of population growth, and by analytical and numerical methods in Barton and Turelli [9] for density-independent models analogous to those considered here. Barton and Turelli (Eq. 32) showed that for an idealized cubic model with an unstable equilibrium 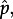 a steady immigration rate greater than2.9
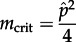
into an initially uninfected population produces local fixation of a variant corresponding to *Wolbachia* that causes complete CI (i.e. *s*_h_ = 1). This provides a point of reference for our numerical results.

First note that if 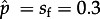 in our CI model, condition (2.9) requires *m* > 0.0225. If instead of the idealized cubic model of Barton & Turelli [9], we use the Caspari & Watson [25] model that we generalized to produce our recursions, the numerically determined value of *m*_crit_ increases to 0.0267, about 19 per cent above prediction (2.9). Hence, as noted by Hancock *et al.* [30], a very low rate of steady release can suffice to produce local population transformation. These analyses ignore the question of how long population transformation takes. Without introducing resistance alleles, *m*_c_ = 0.0471 suffices to produce a 95 per cent infection frequency within 20 generations. This is nearly double the input required without a time constraint, but is still relatively small. If we further stipulate only 10 releases, the required immigration rate becomes *m*_c(10)_ = 0.0563.

**Figure 2.**
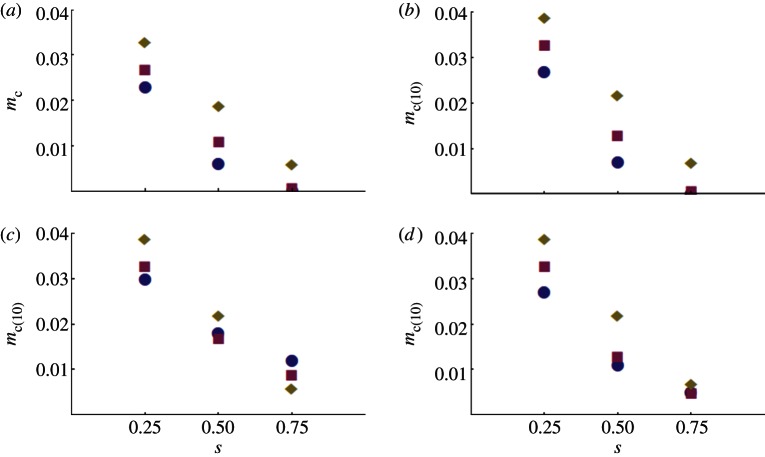
Minimum value of the ‘effective release rate,’ *m*, that produces *Wolbachia* frequency *p_t_* = 0.95 within 20 generations, assuming *r*_I,0_ = 1, and *s*_f_ = 0.3. In each panel, the selection against susceptibility (*s*) varies as does the dominance of susceptibility (*h*: 0.9, diamonds; 0.5, squares; 0.1, circles). (*a*,*b*) Assume no pesticide resistance in the resident population (*r*_U,0_ = 0) and investigate the effects of limiting the releases to the first 10 generations (*b*) versus every generation (*a*). The reference values, without insecticide resistance, are *m*_c_ = 0.0471 for (*a*) and *m*_c(10)_ = 0.0563 for (*b*). (*c*,*d*) Investigate the effects of a low level of insecticide resistance in the target population: *r*_U,0_ = 0.001 (*c*) or *r*_U,0_ = 0.01 (*d*). (Online version in colour.)

Figure 2 shows how *m_c_* and *m_c_*_(10)_ are reduced by introducing resistance alleles along with *Wolbachia*. Panels (*a*,*b*) contrast *m_c_* and *m_c_*_(10),_ whereas panels (*c*,*d*) show the effects on *m_c_*_(10)_ of having a low level of resistance in the pre-release target population. As in figure 1, we vary the dominance of the susceptibility allele (*h*) and the intensity of selection against susceptible homozygotes (*s*) in each panel. The quantitative effects roughly follow those produced by the simpler analysis illustrated in figure 1. If selection for resistance is weaker than the fitness cost imposed by *Wolbachia* (i.e. *s* < *s*_f_, as illustrated by *s* = 0.25 in each panel), introducing resistance with *Wolbachia* has little effect on the minimum introduction rate. By contrast, with strong insecticide-induced selection, the necessary introduction rates can be at least halved (*s* = 0.5) or reduced by a factor of four of more (*s* = 0.75). In comparison to the single-release results illustrated in figure 1, these conclusions seem less sensitive to the dominance of resistance and the presence of low levels of resistance in the target population. As in figure 1, panels (*b*–*d*) show a complex interaction between the intensity of resistance selection (*s*), dominance (*h*) and the initial frequency of resistance in the target population (*r*_U,0_). Again this reflects: (i) the initial rapid fall of resistance among infected individuals as they mate with residents and (ii) the dynamics of the rapid rise of resistance in both the infected and uninfected individuals under insecticide-induced selection when *r*_U,0_ > 0.

## 3. Discussion

The approach outlined above for introducing a *Wolbachia* strain into a mosquito population has three advantages. The first advantage is that it is consistent with existing control options in countries where mosquito-borne diseases are endemic. For instance, fogging and applications of chemicals to breeding sites are commonly used to control *A. aegypti* transmitting dengue [12]; and the approach is, therefore, consistent with ongoing practices that are currently widely and routinely applied. This is particularly important for community engagement because *Wolbachia* releases can then be implemented alongside strategies that are already accepted. These strategies are known to effectively reduce population size and perhaps transmission for short periods in areas where dengue is endemic [e.g. 29,31], although numbers recover again after fogging as immature stages develop and emerge [32].

The second advantage is that the approach can facilitate the introduction of a *Wolbachia* infection while minimizing any increase (or even causing a decrease) in mosquito population size. Population suppression through chemical applications is an integral component of the strategy. Moreover, the strategy results in a lower threshold *Wolbachia* frequency for invasion, so that fewer *Wolbachia*-infected mosquitoes need to be released. This overcomes one of the challenges of the *Wolbachia*-based strategy as implemented recently in Australia [8], where successful introduction depended on community acceptance of a transient increase in the *Aedes* mosquito population.

Finally, the approach can be used to bolster the frequency of *Wolbachia* again should reinvasion by uninfected mosquitoes occur. So for instance if there is partial loss of the infection during the dry season because of poor viability of *Wolbachia*-infected eggs in quiescent state (as in *w*MelPop—[10]), this can be countered by a temporary spraying programme to re-establish the infection until it locally reaches a high frequency. Population suppression using chemical applications might also be used to aid the spread of *Wolbachia* through barriers of high population density (cf. [9]).

To highlight the impact of the strategy on the speed of invasion and mosquito population size, we consider a *Wolbachia* introduction into an area of similar size to those invaded in 2011 (Gordonvale and Yorkeys Knob near Cairns) by *w*Mel [8]. Each area consists of around 600 houses. After 60 days of weekly releases at around 7500 females per week, the infection reached a frequency of around 90 per cent in Yorkeys Knobs and 60 per cent in Gordonvale, which is consistent with a fitness cost of around 20 per cent of the infected mosquitoes (though it was not clear if this reflected costs associated with cage rearing or owing to the presence of the infection). During the release period, the number of *A. aegypti* adults doubled at Yorkeys Knob and increased by 1.5 times at Gordonvale [8]. If an infection with a 40 per cent fitness cost was introduced into these areas over the same period, the expected frequency of infection at 60 days (calculated as outlined by Turelli in the electronic supplementary material to Hoffmann *et al.* [8]) would be 70 per cent at Yorkeys and only 40 per cent in Gordonvale. In contrast, with 50 per cent adult mortality of uninfecteds owing to weekly fogging, complete invasion is predicted based on the above recursions at Yorkeys by 40 days, and the expected frequency at Gordonvale is around 80 per cent after 60 days, with a likely reduction in population size at Gordonvale and a small increase at Yorkeys. Overall, our theoretical results suggest that with plausible levels of insecticide selection, the transformation of the Yorkeys Knob and Gordonvale populations could have been achieved in a comparable time by releasing only half as many mosquitoes. These results highlight that applications of adulticides promote the rapid and localized invasion of infections with substantial fitness costs.

However, a drawback of the approach lies in the introduction of resistance alleles into a population. There are likely to be ethical issues associated with releasing resistance alleles, particularly if these are initially absent or at a low frequency in a population. Because *A. aegypti* is invasive, resistance alleles might also spread from the release area into adjacent regions. If for some reason there is attenuation of the effects of *Wolbachia* on disease transmission, a release programme might have introduced resistance alleles into populations without much benefit (although we have not seen attenuation of *Wolbachia* effects so far in either *w*Mel or *w*MelPop laboratory cultures or field introductions).

Three factors mitigate the uncontrolled spread of resistance alleles. First, these alleles remain associated with the introduced *Wolbachia* infection; the lack of maternal leakage of the infection and complete cytoplasmic incompatibility in *A. aegypti* [10,33] mean that nuclear-resistance alleles are not transferred into the uninfected component of the population. Second, the unstable equilibrium point associated with *Wolbachia* infections would act against R alleles persisting once they have spread to nearby areas. Previous results with the *w*Mel infection have shown that the infection failed to spread outside the release area despite occasional long distance movement into adjacent suburbs [8], and despite the fact that this infection only has a relatively small deleterious fitness effect [1]. Resistance alleles associated with a *Wolbachia* infection with substantial fitness costs would be even less likely to spread than *w*Mel. Third, were attenuation to occur and releases terminated, the association between resistance alleles and *Wolbachia* would be lost quite quickly because matings between infected females and uninfected males are compatible, introducing susceptible alleles into the *Wolbachia*-infected component of a population. The fate of resistance alleles would then be dictated by fitness costs and ongoing selection for resistance—the same processes affecting the frequency of resistance alleles in populations prior to releases taking place.

Nevertheless, it seems prudent to take a cautionary approach when deciding on the nature of resistance alleles to introduce into populations. For instance, lines should not be introduced that have cross resistance to any active compounds likely to be important in current and future chemical control programmes, such as insect growth regulators and Bt toxins. Instead, released lines might carry resistance to chemicals no longer in widespread use (such as carbamates which have been replaced by pyrethroids). Moreover, any release needs to be accompanied by a detailed monitoring programme testing for resistance in target populations and the surrounding area.

In summary, the approach we have developed should facilitate the invasion of *Wolbachia* strains with deleterious effects. However, although in this approach non-native resistance alleles are only ever present in *Wolbachia*-infected individuals, regulatory authorities might be concerned about introducing new alleles that make mosquito control more difficult through conventional means. For this reason, it will be important to weigh the benefits and likely risks of this strategy and undertake careful background monitoring of populations. Deployment of this type of strategy will always require ongoing community engagement and close interaction with authorities, as has been the case with *Wolbachia* introductions [8,34].
